# *Mycobacterium*-Host Cell Relationships in Granulomatous Lesions in a Mouse Model of Latent Tuberculous Infection

**DOI:** 10.1155/2015/948131

**Published:** 2015-05-03

**Authors:** Elena Ufimtseva

**Affiliations:** The Institute of Biochemistry, Siberian Branch of the Russian Academy of Medical Sciences, 2 Timakova Street, Novosibirsk 630117, Russia

## Abstract

Tuberculosis (TB) is a dangerous infectious disease characterized by a tight interplay between mycobacteria and host cells in granulomatous lesions (granulomas) during the latent, asymptomatic stage of infection. *Mycobacterium*-host cell relationships were analyzed in granulomas obtained from various organs of BALB/c mice with chronic TB infection caused by *in vivo* exposure to the Bacillus Calmette-Guérin (BCG) vaccine. Acid-fast BCG-mycobacteria were found to be morphologically and functionally heterogeneous (in size, shape, and replication rates in colonies) in granuloma macrophages, dendritic cells, and multinucleate Langhans giant cells. Cord formation by BCG-mycobacteria in granuloma cells has been observed. Granuloma macrophages retained their ability to ingest damaged lymphocytes and thrombocytes in the phagosomes; however, their ability to destroy BCG-mycobacteria contained in these cells was compromised. No colocalization of BCG-mycobacteria and the LysoTracker dye was observed in the mouse cells. Various relationships between granuloma cells and BCG-mycobacteria were observed in different mice belonging to the same line. Several mice totally eliminated mycobacterial infection. Granulomas in the other mice had mycobacteria actively replicating in cells of different types and forming cords, which is an indicator of mycobacterial virulence and, probably, a marker of the activation of tuberculous infection in animals.

## 1. Introduction

*Mycobacterium tuberculosis* is an infectious agent that causes asymptomatic latent, chronic infection and can provoke active disease in man and animals. At the latent stage of tuberculous infection, mycobacteria can penetrate into organs and tissues and persist there for decades before a possible activation of the tuberculous process followed by the development of active disease [1–4]. Studies of the mechanisms of mycobacterial survival in the host organisms during latent TB infection and the mechanisms of their reactivation and replication are extremely important for the development of new vaccines, medicines, and methods for tuberculosis treatment. These works have since recently become especially important because of the emergence and spread of high-virulence strains of mycobacteria that possess multidrug and extensive drug resistance [5].

As is known, granulomas that form chronic inflammatory lesions and are composed of diverse immune cells, mainly macrophages, are hallmarks of latent tuberculous infection in man and animals [6–9]. Failure, from the side of macrophages, to destroy the absorbed mycobacteria causes a risk of activation and the development of tuberculosis [4, 10, 11]. Although knowledge about the quantity and the functional state of mycobacteria during latent infection is important, this information about mycobacteria in granuloma cells remains insufficient. The bacteriological method, which is generally used for assessing the multiplicity of mycobacterial infection in animal organs and tissues, involves inoculation of their homogenates on special agar media and counting colony-forming units. However, this allows only generalized data on the number of mycobacteria during latent infection to be obtained [12–16]. Neither inspecting mycobacteria on the histological sections of animal tissues [17–20] nor* in vivo* studies of granulomas [21] in the livers of mice infected with BCG, an attenuated live strain of* Mycobacterium bovis,* allow the multiplicity of infection (MOI) in the granuloma cells to be inferred.

In the past decade, information on the state of mycobacteria (i.e., whether they are acid-fast or otherwise) and their metabolic status (i.e., whether they are replicating or otherwise) in cells has been obtained via infecting human and animal cells and cell cultures* in vitro* [22–25]. It has been demonstrated that populations of mycobacteria growing in macrophages and in extracellular environments are morphologically and functionally heterogeneous and contain bacteria with resistance to various drugs [26, 27]. Virulent and attenuated mycobacterial strains behaved differently in* in vitro* cell cultures. For example, the active replication of mycobacteria of only virulent strains was observed, using electron microscopy, both in phagosomes and in the cytoplasm of infected cells within a period of 2 to 7 days following infection* in vitro* [28, 29]. At the same time, BCG and attenuated strains of* M. tuberculosis* have been found only in vacuolar compartments of cells, which is where they were later destroyed before they could start to replicate. After invasion of mouse bone marrow macrophages by a virulent* M. tuberculosis* strain and BCG-mycobacteria* in vitro*, the respective parameters of the transcriptome and gene regulatory networks of mycobacterial genes were different [30].

The number of mycobacteria in granuloma macrophages in acute tuberculous infection after treatment with various antituberculosis drugs was assessed in zebrafish (*Danio rerio*) juveniles infected with* M. marinum* [26, 31]. Cord formation (the indicator of mycobacterial virulence) in zebrafish granulomas was observed exclusively outside cells [31, 32]. On the whole, these studies do not provide a complete picture of relationships between mycobacteria and granuloma cells that contain them. Therefore, knowledge about the exact mycobacterial counts in granuloma cells is essential for the study of tuberculous infection in animal and human organs and tissues both at the latent stage of tuberculosis and during its reactivation.

Infection of mice with* M. tuberculosis* is known to result in a fatal increase in bacterial burden, while the bacterial burden in chronically infected humans is low [33]. By contrast, the bacterial burden following infection of mice with the BCG vaccine is as low as it is observed in latent human infection with* M. tuberculosis*. That is why some researchers consider BCG infection in mice the model of choice for studying latent mycobacterial infection [21, 34, 35].

In this work, we have analyzed the relationships between intracellular mycobacteria and granuloma host cells in mice with latent TB infection. This analysis was performed using an* ex vivo* model of monolayer granuloma culture samples obtained from spleens, lungs, and bone marrow of mice infected with the BCG vaccine* in vivo* [9]. In a result, we assessed the functional state of the mycobacteria and their number in granuloma cells of various types from different organs of the mice. It was ascertained that these granuloma cells contained single BCG-mycobacteria and colonies resulting from replication, often in the same host cell. We have for the first time observed the formation of cords by replicating mycobacteria, presumably in the cytoplasm of granuloma macrophages and dendritic cells. Our study indicates that BCG-mycobacteria in granuloma cells obtained from various organs of mice with chronic TB infection are functionally heterogeneous. The mice also differed in the number of BCG-containing cells in granulomas they had, which may indicate a difference in the mice's infection status during the latent period of disease.

## 2. Materials and Methods

### 2.1. Animals

Two-month-old BALB/c male mice were obtained from the Animal Breeding Facility of the Institute of Cytology and Genetics of the Siberian Branch of the Russian Academy of Sciences (Novosibirsk, Russia). Mice were bred and maintained under standard vivarium conditions, with water and food provided ad libitum. Animal experiments were conducted in accordance with “The Guidelines for Manipulations with Experimental Animals” issued by the Russian Ministry of Health (guideline 755). All experimental procedures were approved by the Local Ethical Committee of the Institute of Biochemistry, SB RAMS, Novosibirsk, Russia.

### 2.2. Infection of Mice

Mice were infected with a vaccine prepared from an attenuated live strain of* M. bovis* (the Bacillus Calmette-Guérin vaccine, BCG-1, Allergen, Stavropol, Russia) at a dose of 0.5 mg per mouse, which amounted to 3 × 10^6^ viable BCG-mycobacteria in 0.9% NaCl solution. Seventeen mice were each infected via tail vein injection with 100 *μ*L of the suspension and four mice were each infected intraperitoneally with 200 *μ*L of the suspension.

### 2.3. Isolation and* Ex Vivo* Culture of Mouse Granulomas

Mice were anesthetized and sacrificed by cervical dislocation. Isolation of granulomas from the spleens, lungs, and bone marrow of mice after 20 days, one month, and two months following infection was performed as previously described [9]. Bone marrow was flushed from femur bones with RPMI 1640 (BioloT, St. Petersburg, Russia). Granulomas in the RPMI 1640 growth medium containing 10% heat-inactivated fetal bovine serum, 2 mM glutamine, and 50 *μ*g/mL gentamicin (BioloT, St. Petersburg, Russia) were placed at low-medium density to 24-well tissue culture plates (Orange Scientific, Belgium) with glass coverslips at the bottom and cultured in 0.5 mL medium for several days at +37°C in an atmosphere containing 5% CO_2_. Granulomas were isolated from mice 1 and 2 on day 20 following intraperitoneal infection; mice 1 and 3 ÷ 5 after one month following intraperitoneal and intravenous infection, respectively; mice 4 ÷ 6, 8 ÷ 10, 12 ÷ 14, 16, and 21 ÷ 24 after two months following intravenous infection; and mouse 25 after two months following intraperitoneal infection. Peritoneal macrophages were isolated from mouse 2 after 20 days and mouse 25 after two months following intraperitoneal infection and cultured under the same conditions as the granuloma cells. After isolation of granulomas from the femur bones of mice 1 and 2 after 20 days following infection, the other bone marrow cells were cultured under the same conditions as the granuloma cells.

### 2.4. Cell Staining

After 2–5 days of* ex vivo* culture, granuloma cells and peritoneal and bone marrow macrophages on coverslips were fixed with 4% formaldehyde solution in phosphate buffer saline (PBS, pH 7.4) for 10 minutes at room temperature. To visualize acid-fast bacteria, the preparations were washed with PBS and stained after Ziehl-Neelsen. The cells were further counterstained with 1% methylene blue. In the experiments using LysoTracker Red DND-99 (Invitrogen, USA, L7528), the cell preparations were incubated with 50 nM of the acidotropic dye for 5 minutes at +37°C in 5% CO_2_ before fixation. The cell preparations were fixed as described above, washed with PBS, permeabilized within 2 minutes in 0.3% Triton-X100 solution, blocked in PBS containing 2% BSA, and finally incubated first with rabbit polyclonal primary antibodies to mycobacteria (Abcam, England, ab20832) diluted 1 : 200 and then with Alexa 488-conjugated goat anti-rabbit Ig secondary antibodies (Invitrogen, USA, A11034) diluted 1 : 400. Some of the fixed cell preparations were washed with PBS, blocked in PBS solution containing 2% BSA, and incubated with rat primary antibodies to mouse CD1d (BD Pharmingen, USA, 553843) or hamster antibodies to mouse CD80 labeled by PerCP-Cy 5.5 (BD Pharmingen, USA, 560526) diluted 1 : 50 and 1 : 100, respectively. Then, these preparations were washed with PBS, treated within 2 minutes in 0.3% Triton-X100 solution, and incubated with hamster primary antibodies to mouse IL-1*α* (BD Pharmingen, USA, 550604) or rat primary antibodies to mouse IFN*γ* (BD Pharmingen, USA, 559065) diluted 1 : 50 and 1 : 100, respectively. Fluorescent visualization of the proteins was enabled using goat polyclonal FITC-labeled antibodies against rat IgG (Abcam, England, ab6266) diluted 1 : 400. The other cell preparations were incubated with mouse monoclonal antibodies against hamster IgG (BD Pharmingen, USA, 550335) diluted 1 : 50 and then with Alexa 555-conjugated goat anti-mouse IgG secondary antibodies (Invitrogen, USA, A1422) diluted 1 : 400. The cell preparations were incubated with the appropriate antibodies for 60 minutes at room temperature. Fluorescent staining was analyzed using the VECTASHIELD Mounting Medium with DAPI (4′,6-diamidino-2-phenylindole) (Vector Laboratories, USA, H-1200). The confocal images of the cells were recorded; the preparations were washed from VECTASHIELD Mounting Medium in PBS for 20 minutes and restained for acid-fast mycobacteria using the Ziehl-Neelsen stain.

### 2.5. Microscopy

The cytological preparations were examined at the Shared Center for Microscopic Analysis of Biological Objects of the Institute of Cytology and Genetics, SB RAS, using an Axioscop 2* plus* microscope (Zeiss) and objectives with various magnifications (Zeiss), and photographed using an AxioCam HRc camera (Zeiss); the images were analyzed using the AxioVision 4.7 microscopy software (Zeiss). Cell preparations were stained with fluorescent dyes and examined under an LSM 780 laser scanning confocal microscope (Zeiss) using the LSM Image Browser and ZEN 2010 software (Zeiss).

### 2.6. Statistical Analysis

Statistical data processing was performed using MS Excel 2007 (Microsoft). Differences were tested for significance using Student's* t*-test.

## 3. Results

### 3.1. BCG-Mycobacteria in Mouse Granuloma Cells

Granulomas from spleens (S/) and bone marrow (BM/) were isolated on day 20 (/20 d) following infection with BCG vaccine (this is how long it takes mice to develop adaptive immunity to BCG [21]), and granulomas from spleens and lungs (L/) were isolated after one month (/1 m) and two months (/2 m) following infection. All the granulomas were seeded into culture plates. The granulomas isolated after 20 days, one month, and two months following infection were denoted as Gran/20 d, Gran/1 m, and Gran/2 m, respectively. Because none of the mice had been observed to have acute tuberculous infection at the time of granuloma isolation, it was concluded that these granulomas were isolated at the latent, chronic stage of BCG infection. Monolayer cultures of cells that had migrated from each granuloma were obtained. The cellular composition of each granuloma isolated from mice after one month and two months following infection, the expression of leukocyte surface markers, and the production of proinflammatory cytokines and growth factors by granuloma cells had been characterized previously [9]. Any of the monolayer cultures of granuloma cells that may or may not retain cell clusters in the center of granulomatous lesions will be referred to as the “granuloma” throughout. Diffuse leukocyte infiltrates were obtained mainly from the spleen of mouse 6 S/2 m. Very few granulomas (or none at all) were isolated from each mouse lung. Only a few were obtained from bone marrow on day 20 following infection and many, from the spleens. All granulomas from mice 1/20 d and 2/20 d were solid and contained predominantly macrophages, dendritic cells (approximately 10% of the granuloma macrophage population), and a varying number of lymphocytes. Fibroblasts and neutrophils were occasionally observed in Gran/20 d granulomas; however, eosinophils, megakaryocytes, or multinucleate Langhans giant cells were not.

**Figure 1:**
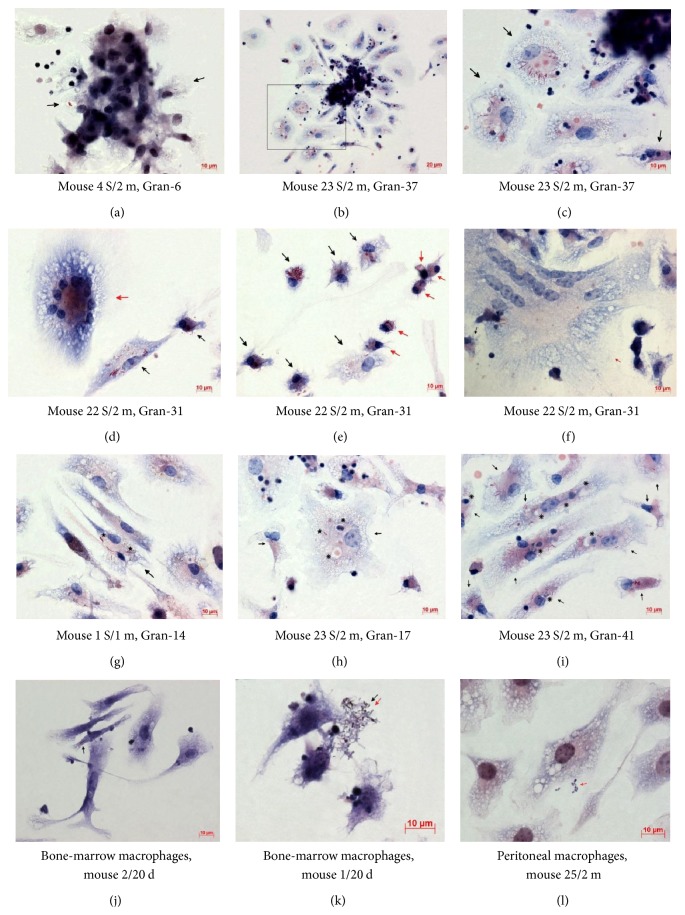
((a)–(i)) BCG-mycobacteria in the cells of granulomas (Gran) obtained from spleens (S/) of mice after one month (/1 m) and two months (/2 m) following infection with BCG* in vivo* and after* ex vivo* culture for several days. ((a) and (c)–(j)) Infected macrophages (black arrows) and (e) infected dendritic cells (red arrows). ((a) and (b)) Granulomas with migrating cells. (c) A close-up of the granuloma fragment in the black frame in (b). ((d)–(i)) Fragments of granulomas. ((d) and (f)) Infected multinucleate Langhans giant cells (red arrows). ((g) and (h)) Replicating BCG-mycobacteria in macrophage vacuoles are indicated by black asterisks. ((h) and (i)) Vacuoles with debris of lymphocytes and thrombocytes in infected macrophages are indicated by black snowflakes. ((k) and (l)) Acid-fast (black arrows) and nonacid-fast (red arrows) mycobacteria outside bone marrow and peritoneal macrophages obtained from mice after 20 days (/20 d) and two months (/2 m) following infection with BCG* in vivo*. Acid-fast BCG-mycobacteria after Ziehl-Neelsen staining. Scale bars: 20 *μ*m (b) and 10 *μ*m ((a) and (c)–(l)).

We determined, with the use of staining after Ziehl-Neelsen, the exact number and the functional state of acid-fast BCG-mycobacteria (which by definition have undamaged cell walls) in granuloma cells isolated from various organs of mice with latent TB infection. BCG-mycobacteria were mainly found in macrophages and rarely in dendritic cells (Figures 1(a)–1(i)). Langhans giant cells, too, contained BCG-mycobacteria (Figures 1(d) and 1(f)). No acid-fast BCG-mycobacteria were found in lymphocytes, fibroblasts, eosinophils, or megakaryocytes. After a thorough examination of 295 neutrophils in granulomas from mice and 288 neutrophils in the leukocyte infiltrates from mouse 6 S/2 m, BCG-mycobacteria were found only in two neutrophils, one from mouse 8 S/2 m and one from mouse 6 S/2 m. No acid-fast mycobacteria were found in the peritoneal macrophages of the mice that had not been exposed to BCG; however, such BCG-mycobacteria were found in 2.38% of a total of over 9000 peritoneal macrophages assayed from mouse 2/20 d and in 0.11% of a total of over 18,000 peritoneal macrophages assayed from mouse 25/2 m. Acid-fast mycobacteria were also found in 0.13% of a total of over 9000 bone marrow macrophages assayed from mouse 1/20 d and in 0.05% of a total of over 18,000 bone marrow macrophages assayed from mouse 2/20 d (Figure 1(j)). Each peritoneal and bone marrow macrophage had one* Mycobacterium* on most occasions. Dendritic cells in the cultures of peritoneal and bone marrow macrophage did not contain BCG-mycobacteria. Interestingly, some acid-fast and some nonacid-fast BCG-mycobacteria were found in extracellular locations in all tissue cultures of peritoneal and bone marrow macrophages isolated from mice on day 20 following infection (Figure 1(k)) and in the culture of peritoneal macrophages isolated from mouse 25 after two months following infection* in vivo* (Figure 1(l)).

BCG-mycobacteria observed in granuloma cells in the* ex vivo* culture were coccid- or rod-shaped and varied in size from 0.5 to 8 *μ*m (Figures 1(a)–1(i)). The smallest acid-fast mycobacteria were mainly found in macrophages of granulomas and the tissue cultures checked on day 20 following infection and in the peritoneal macrophages of mouse 25/2 m. The granulomas contained cells with a varying number of BCG-mycobacteria in them (Figures 1(a) and 1(c)–1(i)). Most granuloma macrophages each contained a single BCG-mycobacterium. At the same time, granulomas with macrophages each containing two or more BCG-mycobacteria were common. We observed BCG-mycobacteria paired in a V-shaped manner (Figures 1(d), 1(e), 1(g), and 1(h)). Mycobacteria are known to take on a V-shape during the late stages of cell division [36–38]. BCG-mycobacteria were detected near the nuclei and close to the cell membrane in different parts of the cells. Acid-fast mycobacteria were found both within visible membrane-bound vacuoles (the places where BCG-mycobacteria have sometimes been observed to grow) (Figures 1(a) and 1(g)-1(h)) and presumably in the cytoplasm of the macrophages, or so the lack of visible membrane structures around replicating microorganisms in large colonies suggests (Figures 1(c)–1(e) and 1(i)). However, a more detailed examination of granulomas' cellular compartments containing mycobacteria using antibodies against different endosomes and other cellular markers to characterize them at various stages of maturation, differentiation, and functional state appears to be necessary. BCG-mycobacteria were found either as single cells or as clusters of two or more replicating microorganisms (Figures 1(c)–1(j)) in the macrophages of all types and sizes (Figures 1(d) and 1(e)). Infected macrophages were found both at the periphery and in the center of granulomas in* ex vivo* culture. It is noteworthy that the number of BCG-mycobacteria in macrophages did not affect the ability of granuloma cells to migrate from solid granulomas in monolayer cell cultures* ex vivo*. The macrophages that had more than 30 BCG-mycobacteria in each and had active front edges of migrating cells were found at the periphery of the granulomas, and so were the macrophages that contained single or no BCG-mycobacteria (Figures 1(b) and 1(c)). It has been shown in zebrafish that, at the initial stage of infection with* M. marinum*, the more mycobacteria macrophages contain, the less mobile they are [39].

### 3.2. The Number of Granulomas and Granuloma Cells with BCG-Mycobacteria in Mice

**Figure 2:**
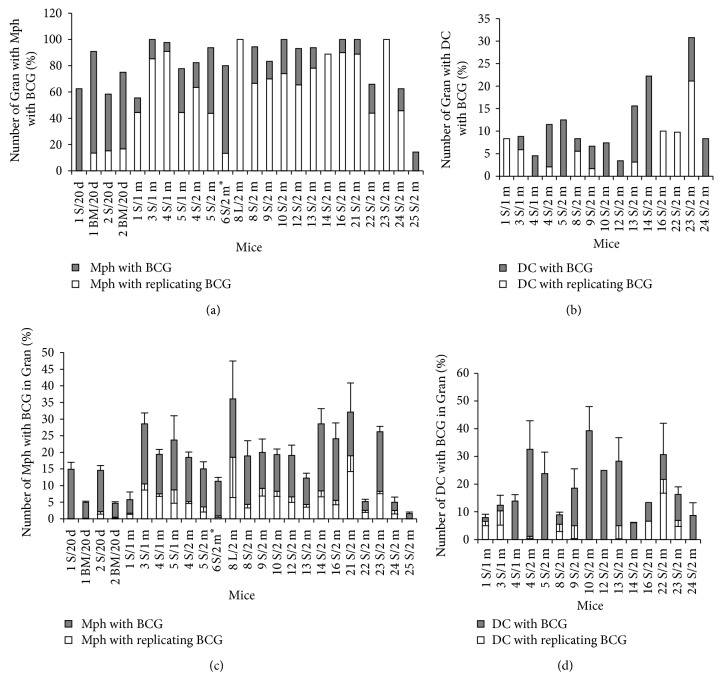
(a–d) Granulomas (Gran) obtained from the lungs (L/), spleens (S/), and bone marrow (BM/) of mice after 20 days (/20 d), one month (/1 m), and two months (/2 m) following infection with BCG* in vivo* and after* ex vivo* culture for several days, in which macrophages (Mph) or dendritic cells (DC) with acid-fast BCG-mycobacteria (BCG) in them were counted. ((a) and (b)) The figures represent the total number of granulomas with infected Mph and DC and the number of granulomas with Mph and DC with colonies of replicating BCG-mycobacteria expressed as a percentage of the total number of granulomas inspected. ((c) and (d)) The number of Mph (and DC) with any BCG (single or as colonies) and with colonies of replicating BCG, both expressed as a percentage of the total number of granuloma Mph (and DC). Data are expressed as the means ± SEM. (∗) Leukocyte infiltrates from mouse 6 S/2 m.

For each of *n* granulomas assayed (1 S/20 d, *n* = 32; 2 S/20 d, *n* = 72; 1 BM/20 d, *n* = 22; 2 BM/20 d, *n* = 12; 1 S/1 m, *n* = 36; 3 S/1 m, *n* = 34; 4 S/1 m, *n* = 44; 5 S/1 m, *n* = 9; 4 S/2 m, *n* = 96; 5 S/2 m, *n* = 16; 8 L/2 m, *n* = 2; 8 S/2 m, *n* = 36; 9 S/2 m, *n* = 60; 10 S/2 m, *n* = 27; 12 S/2 m, *n* = 29; 13 S/2 m, *n* = 32; 14 S/2 m, *n* = 9; 16 S/2 m, *n* = 10; 21 S/2 m, *n* = 9; 22 S/2 m, *n* = 41; 23 S/2 m, *n* = 52; 24 S/2 m, *n* = 24 and 25 S/2 m, *n* = 21), we provide the number of infected cells, the number of BCG-mycobacteria in these cells, and the standard errors of the mean (SEM). Data reflecting the numbers of granulomas containing macrophages and dendritic cells with replicating and nonreplicating BCG-mycobacteria as well as the numbers of such macrophages and dendritic cells in granulomas from mice are shown in Figures 2(a)–2(d). The mice differed in the number of splenic granulomas containing macrophages with BCG-mycobacteria they had and in the number of splenic granulomas containing macrophages with replicating BCG-mycobacteria they had (Figure 2(a)). The variance for the number of splenic granulomas containing macrophages with BCG-mycobacteria ranged from 14% (mouse 25 S/2 m) to 100% (mice 3 S/1 m, 10 S/2 m, 16 S/2 m, 21 S/2 m, and 23 S/2 m). Most granulomas had cells with replicating BCG-mycobacteria (Figure 2(a)). Splenic and bone marrow granulomas differed in the number of macrophages containing BCG-mycobacteria, single or replicating, they had (Figure 2(c)). Interestingly, granulomas containing small numbers of macrophages but large numbers of BCG-mycobacteria in them and granulomas containing large numbers of macrophages with a very few cells with acid-fast mycobacteria in them were observed in the same mice. The average number of macrophages with BCG-mycobacteria in granulomas varied from 1.5–5% (mice 1 BM/20 d, 2 BM/20 d, 1 S/1 m, 22 S/2 m, 24 S/2 m, and 25 S/2 m) to 25–30% (mice 3 S/1 m, 14 S/2 m, 21 S/2 m, and 23 S/2 m) of the total number of macrophages in the granulomas. Infected macrophages in splenic granulomas from most mice made up from 15% to 20% on most occasions. It is noteworthy that a large number of bone marrow granulomas from mice 1/20 d and 2/20 d had macrophages with acid-fast BCG-mycobacteria; however, the total number of infected macrophages was lower in them than in the splenic granulomas from the same mice. On the whole, granulomas, in which all macrophages had acid-fast BCG-mycobacteria, were not found.

Granulomas with infected dendritic cells in them were not identified in all BCG-infected mice. Additionally, such granulomas were fewer (6–15%) than those with infected macrophages in them (Figure 2(b)). A larger number of splenic granulomas (some 30%) with BCG-containing dendritic cells were found only in mouse 23 S/2 m. The number of infected dendritic cells varied substantially in granulomas from different mice, with the largest number being determined in the splenic granulomas from mice 4 S/2 m, 10 S/2 m, 13 S/2 m, and 22 S/2 m (Figure 2(d)). Most granuloma dendritic cells contained from one to three acid-fast mycobacteria. BCG-mycobacteria were observed either as single cells or as groups of replicating microorganisms presumably in the cytoplasm of dendritic cells (Figure 1(e)), because no visible vacuoles with mycobacteria were resolved in those cells. However, the intracellular compartments where mycobacteria reside in mouse granuloma dendritic cells remain to be identified. It is noteworthy that Jiao and colleagues [40] found no evidence for BCG replication in dendritic cells isolated from spleens of mice after 12 days following infection with BCG* in vivo*. It is possible that the difference in the observations is due to the difference in the status of dendritic cells at the stages of acute and latent BCG infection in mice. However, no dendritic cells with BCG-mycobacteria in them were found in the leukocyte infiltrates of mouse 6 S/2 m, which was assayed presumably during acute TB infection, as they had an increased number of neutrophils [9]. No acid-fast mycobacteria were found in the dendritic cells of splenic granulomas isolated from mice on day 20 following infection* in vivo*. A single mycobacterium was observed in a single dendritic cell within a single bone marrow granuloma from mouse 1/20 d.

Furthermore, we found BCG-mycobacteria in some multinucleate Langhans giant cells in splenic granulomas from different mice (Figures 1(d) and 1(f)). For example, in some granulomas from mouse 22 S/2 m, we observed Langhans giant cells with 7 nuclei, 10 nuclei (Figure 1(d)), and 42 nuclei (Figure 1(f)) containing one, 23, and 11 mycobacteria, respectively, both as single cells and as colonies of replicating mycobacteria. No acid-fast BCG-mycobacteria were found in Langhans giant cells with 6, 9, or 15 nuclei in other granulomas from the same mouse. It is very likely that BCG occurred in the Langhans giant cells as a result of fusion of infected macrophages (Figure 1(f)); however, this assumption has yet to be verified.

### 3.3. The Number of BCG-Mycobacteria in Mouse Granuloma Macrophages

**Figure 3:**
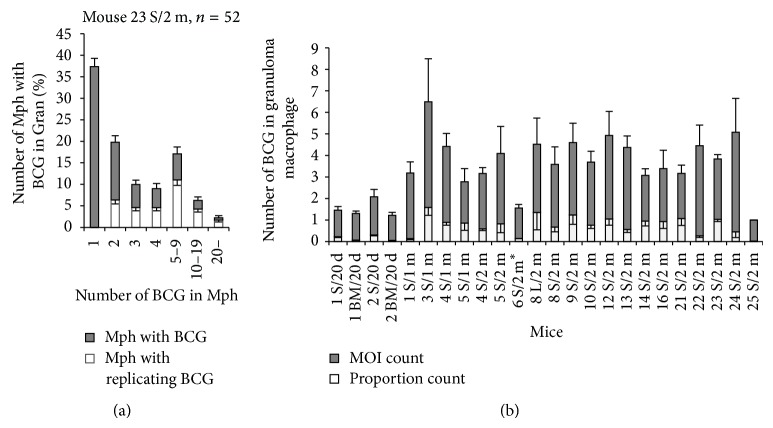
(a) Macrophages (Mph) with different numbers of BCG-mycobacteria (BCG) in the granulomas from mouse 23 S/2 m. Dark bars represent the number of Mph with different BCG numbers calculated as a percentage of the total number of infected Mph in the granulomas. White bars represent the number of Mph containing colonies of replicating BCG-mycobacteria calculated as a percentage of the total number of infected Mph in granulomas. (b) The number of BCG per macrophage in granulomas from different mice calculated as the number of BCG in macrophages relative the total number of infected macrophages (dark bars) or the total number of all granuloma macrophages (white bars) (see text for details). Data are expressed as the means ± SEM. Abbreviations as in the legend to Figure 2. (∗) Leukocyte infiltrates from mouse 6 S/2 m.

The number of BCG in macrophages varied within and between granulomas isolated from the same mouse. Furthermore, they varied between granulomas isolated from different mice. Macrophages with from one to up to 100 BCG-mycobacteria in each, either as single cells or as colonies of replicating mycobacteria (Figures 1(a) and 1(c)–1(i)), were observed, macrophages with two or more BCG-mycobacteria in each being less frequent than those with one mycobacterium in each (Figure 3(a)). Meanwhile, cells with a larger number of mycobacteria (30 or more) were observed in many splenic granulomas, but these cells were rare (Figure 3(a)). To determine the number of BCG-mycobacteria in the mouse granulomas, we used the following two BCG counts. One was calculated as follows: for each granuloma in a mouse, the total number of BCG-mycobacteria in all infected macrophages was determined and divided by the total number of these infected macrophages; the quotients were summed and their sum was divided by the total number of granulomas examined in that mouse. In fact, this count represents the MOI in the granuloma macrophages, and so it will be referred to as the “MOI count” throughout. The other was calculated as follows: for each granuloma in a mouse, the total number of BCG-mycobacteria in all infected macrophages was determined and divided by the total number of macrophages (both infected and not); the quotients were summed and their sum was divided by the total number of granulomas examined in that mouse. This count reflects the proportion of infected granuloma cells, and so it will be referred to as the “proportion count” throughout (Figure 3(b)). These counts differed across the mice. For example, in mouse 3 S/1 m granulomas, both counts were elevated, which was indicative of a higher MOI in granuloma cells on the one hand and, on the other hand, of a high proportion of infected cells in granuloma macrophages. By contrast, in mice 22 S/2 m, the MOI count was elevated and the proportion count was considerably decreased, which implied that fewer granuloma macrophages were infected, while individual granuloma cells had a high bacterial load. It is noteworthy that macrophages in the granulomas from the lung and the spleen of mouse 8/2 m and from the bone marrow and the spleens of mice 1/20 d and 2/20 d had similar MOI counts and similar proportion counts (Figure 3(b)).

### 3.4. Lack of Colocalization of BCG-Mycobacteria and LysoTracker Dye in Mouse Granuloma Macrophages Producing the Proinflammatory Cytokines IFN*γ* and IL-1*α*

Macrophages in the splenic granulomas obtained from all mice had an enhanced ability for phagocytosis and destruction of damaged granuloma cells, mainly lymphocytes and thrombocytes and sometimes neutrophils, in the* ex vivo* culture. Phagosomes with engulfed lymphocytes and thrombocytes at various stages of degradation existed side by side with the vacuoles with acid-fast BCG-mycobacteria in granuloma macrophages (Figures 1(h) and 1(i)). It is noteworthy that no phagosomes with absorbed destroyed cells were observed in the dendritic cells.

**Figure 4:**
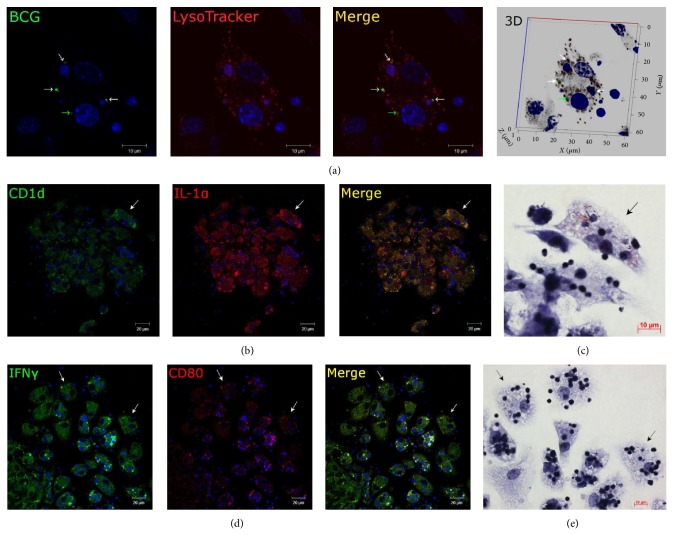
Representative confocal fluorescent images of granuloma macrophages stained for different markers. Fragments of splenic granulomas from mouse 2 after 20 days following BCG infection. Nuclei are stained by DAPI (blue signal). Scale bars: 10 *μ*m (a, c, e) and 20 *μ*m (b, d). (a) Mouse granuloma macrophages stained by the LysoTracker Red DND-99 dye (red signal) and mycobacterial LAM-specific antibodies (green signal) show lack of colocalization of BCG-mycobacteria and host cell lysosomes (lack of yellow signal). A single BCG-mycobacterium (green arrow) and replicating BCG-mycobacteria (white arrows). ((b) and (d)) Immunofluorescent localization of CD1d (green signal) and IL-1*α* (red signal) and IFN*γ* (green signal) and CD80 (red signal) in granuloma cells, respectively. Colocalization of the markers on the confocal images of cells (yellow signal). ((c) and (e)) The same granuloma fragments as in ((b) and (d)) restained for acid-fast BCG-mycobacteria by the Ziehl-Neelsen method. The same macrophages with replicating BCG-mycobacteria and producing proinflammatory cytokines and leukocyte surface markers are indicated by white arrows on the fluorescent ((b) and (d)) images and by black arrows on the Ziehl-Neelsen ((c) and (e)) images.

We used the acidophilic fluorescent LysoTracker Red DND-99 probe to see if BCG-mycobacteria and acidic compartments of splenic and bone marrow granuloma cells as well as peritoneal and bone marrow macrophages from mice 1/20 d and 2/20 d show colocalization. The LysoTracker dye was used at a very low concentration in the cell-incubating medium only for 5 minutes before cell fixation in order to stain mainly lysosomes in the cells [41]. As a result, no colocalization of the acidophilic LysoTracker probe and BCG-mycobacteria detected by antibodies reacting with the major mycobacterial cell wall component glycolipid lipoarabinomannan (LAM) was observed (Figure 4(a)). Therefore, all LAM-labeled mycobacteria had avoided host killing in lysosomes and survived within the granuloma, peritoneal, and bone marrow macrophages of mice after 20 days following infection with BCG* in vivo*.

Our study of the proinflammatory cytokines IFN*γ* and IL-1*α*, bacterial lipids, the glycolipids-presenting molecule CD1d and the costimulatory molecule CD80 demonstrated a significant activation of granuloma cells, but not the peritoneal macrophages, from mice 1/20 d and 2/20 d through a higher expression of these markers regardless of mycobacterial load in the host cells (Figures 4(b) and 4(d)). It is noteworthy that the cytokine-producing granuloma macrophages with increased microbicidal potential too contained replicating acid-fast BCG-mycobacteria in Figures 4(b)–4(e). This result is similar to the previously observed significant increase in the production of proinflammatory cytokines IFN*γ* and cell-associated IL-1*α*, the growth factors GM-CSF and FGFb, the phagocytic receptors CD11b, CD11c, CD14, and CD16/CD32, and the costimulatory molecules CD80, CD83, and CD86 in granuloma cells (with and without acid-fast BCG-mycobacteria) of mice after one and two months following infection with BCG vaccine* in vivo* [9].

Thus, we observed in the* ex vivo* culture of granuloma macrophages that, on the one hand, these macrophages retained their function to ingest engulfed granuloma cells (lymphocytes and thrombocytes) in the host's phagosomes and, on the other hand, that the host cells with increased production of the proinflammatory cytokines IFN*γ* and IL-1*α* had a reduced bactericidal capacity. This allowed BCG-mycobacteria to survive and actively replicate in mouse granuloma cells.

### 3.5. The Cord Morphology of BCG Growth in Mouse Granuloma Cells

**Figure 5:**
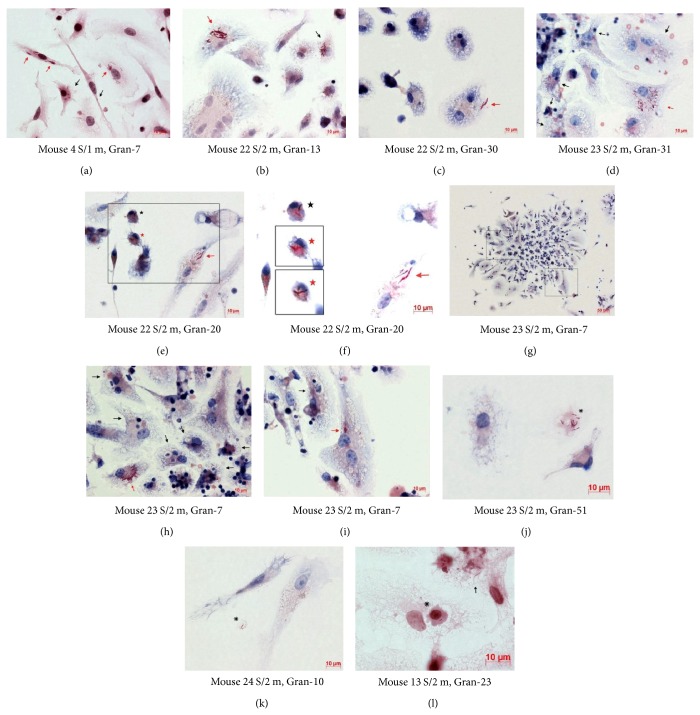
((a)–(i)) BCG cords in the cells of granulomas (Gran) obtained from the spleens (S/) of mice after one month (/1 m) and two months (/2 m) following infection with the BCG vaccine* in vivo* and after* ex vivo* culture for several days. ((a)–(f) and (l)) Macrophages with BCG-mycobacteria (black arrows) and cords (red arrows). ((e) and (f)) Dendritic cells with BCG-mycobacteria (black asterisks) and BCG cords (red asterisks). (f) A high-contrast photo (e) of BCG-mycobacteria and BCG cords in the cytoplasm of dendritic cells. The same dendritic cell at different levels of sharpness is shown in the black frames. ((j) and (k)) BCG-mycobacteria outside granuloma cells (black snowflakes). (l) The only case when a granuloma macrophage had a vacuole (a black asterisk) with an apoptotic cell containing a mycobacterium. ((a)–(f) and (j)–(l)) Fragments of granulomas. ((h) and (i)) Close-ups of granuloma fragments in the black frames in (g). Acid-fast BCG-mycobacteria after Ziehl-Neelsen staining. Scale bars: 50 *μ*m (g) and 10 *μ*m ((a)–(f) and (h)–(l)).

**Figure 6:**
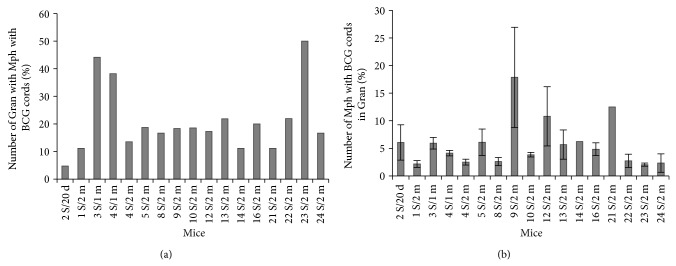
(a) The number of granulomas with Mph containing BCG cords expressed as a percentage of the total number of granulomas assayed. (b) The number of Mph with BCG cords in granulomas from different mice expressed as a percentage of the total number of macrophages in granulomas with Mph containing BCG cords. Data are expressed as the means ± SEM. Abbreviations as in the legend to Figure 2.

Cording is a characteristic morphology of mycobacterial growth on solid and in liquid media, referring to the occurrence of colonies, in which the bacteria are arranged in the form of plaits, bundles, ropes, or cords, while drawing up in a line along their long axes and in close parallel arrangement. Previous studies [42, 43] provide evidence that the cord phenotype of mycobacterial growth correlates with the virulence of mycobacteria. It has been assumed that the cord morphology of mycobacteria is determined by the components of their cell walls and, first of all, by the glycolipids of trehalose-6,6′-dimycolate and its modifications [12, 44–46]. In zebrafish infected with* M. marinum*, cord formation by mycobacteria was for the first time observed in granulomas of juveniles* in vivo* and only in mycobacteria that were growing outside cells [31, 32]. It was for that reason proposed that cord formation is an attribute of the growth of only extracellular bacteria, no matter whether* in vivo* or* in vitro* [30, 31]. Our analysis of cells with acid-fast BCG-mycobacteria in splenic granulomas in* ex vivo* culture proved the presence of BCG cords in the cytoplasm of macrophages and dendritic cells (Figures 1(c)–1(e), 4(b) and 5(a)–5(f)). No visible membrane structures were resolved around the BCG cords in most granuloma cells (Figures 5(a)-5(b), 5(e)-5(f) and 5(h)); however, special studies are required for exploring the intracellular compartments available to the replicating mycobacteria in the mouse cells. Although BCG cords were found only in few granuloma macrophages (from 2% to 18% of infected macrophages in all granulomas), they were found in a large number of splenic granulomas of almost all of the mice, no matter what route of administration was used, and at all checkpoints following infection (Figures 4(b), 5(a)–5(i) and 6(a)-6(b)). By contrast, no extracellular growth of BCG-mycobacteria in granulomas was observed in* ex vivo* culture. Furthermore, no BCG-mycobacteria were observed outside granuloma cells. BCG-mycobacteria were occasionally found in apoptotic bodies and in fragments of the cell cytoplasm located in phagosomes of macrophages (Figure 5(l)) or outside cells (Figures 5(j) and 5(k)).

BCG cords were also observed in some dendritic cells (Figures 5(e) and 5(f)) in several granulomas from mice 9 S/2 m, 22 S/2 m, and 23 S/2 m. These mice had an increased number of granulomas containing macrophages with BCG cords. It is noteworthy that no cords were observed in macrophages or dendritic cells of leukocyte infiltrates from the spleen of mouse 6 S/2 m.

The number of BCG-mycobacteria in the cords in mouse granuloma macrophages and dendritic cells varied from 5 to more than 30 and was from 10 to 20 on most occasions. It is noteworthy that BCG colonies with cords and acid-fast mycobacteria replicating in irregular clumps were often observed simultaneously in the same cell in different mouse granulomas (Figures 1(c)–1(i) and 5(a)-5(b), 5(d)–5(f), and 5(h)-5(i)).

### 3.6. BCG-Mycobacteria in Mouse Spleen Granuloma Cells in Latent Tuberculosis

**Figure 7:**
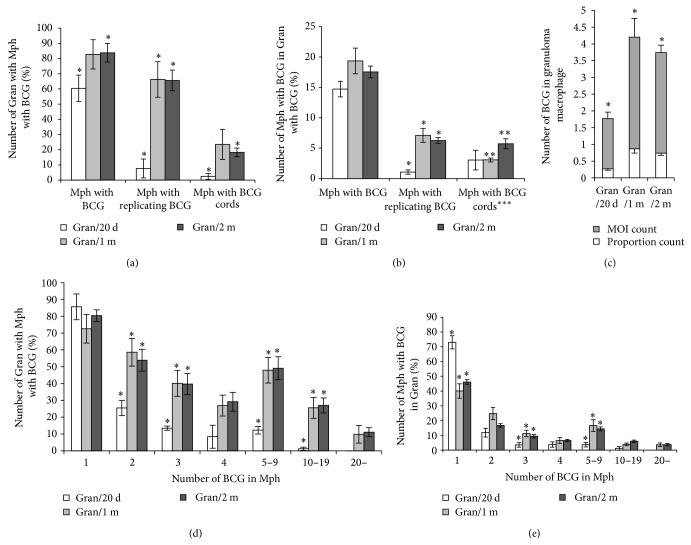
A comparison of the granulomas (Gran) isolated from mouse spleens after 20 days (Gran/20 d), one month (Gran/1 m), and two months (Gran/2 m) following infection with the BCG vaccine* in vivo* and several days in* ex vivo* culture. (a) The number of Gran containing macrophages (Mph) with acid-fast BCG-mycobacteria (BCG) is expressed as a percentage of the total number of Gran assayed. (b) The number of infected macrophages expressed as a percentage of the total number of macrophages in Gran with infected macrophages or (∗ ∗ ∗) in Gran with Mph containing BCG cords. (c) The number of BCG per granuloma macrophage calculated as the number of BCG in the macrophages relative to the total number of infected granuloma macrophages (dark bars) or the total number of granuloma macrophages (white bars) (see text for details). (d) The number of Gran containing Mph with different BCG numbers expressed as a percentage of the total number of Gran with infected Mph. (e) The number of macrophages with different numbers of BCG in Gran expressed as a percentage of the total number of infected macrophages in granulomas. Data are expressed as the means ± SEM. ^∗^ *P* < 0.05 (Gran/20 d versus Gran/1 m or Gran/20 d versus Gran/2 m), ^∗∗^ *P* < 0.01 (Gran/1 m versus Gran/2 m). Data relate to (a) Gran/20 d (2 mice, *n* = 104), Gran/1 m (4 mice, *n* = 123), Gran/2 m (14 mice, *n* = 462), and ((b)–(e)) Gran/20 d (2 mice, *n* = 62), Gran/1 m (4 mice, *n* = 104), and Gran/2 m (14 mice, *n* = 386).

It was found that the number of granulomas containing both macrophages with BCG-mycobacteria and macrophages with colonies of replicating mycobacteria in splenic granuloma cells had changed in the course of the latent TB infection (Figure 7(a)). Some increase in the number of granulomas containing macrophages with acid-fast mycobacteria was observed after 20 days, one month, and two months following infection; however, a considerable increase in the number of splenic granulomas with replicating microbes and BCG cords in macrophages was observed after one month and two months following infection (^∗^ *P* < 0.05, Figure 7(a)). The number of granuloma macrophages with replicating mycobacteria was increased considerably in Gran/1 m and Gran/2 m granulomas as compared with Gran/20 d granulomas (^∗^ *P* < 0.05, Figure 7(b)). No statistically significant change in the number of macrophages with cords was found between granulomas after 20 days, one month, or two months after infection (Figure 7(b)). However, there was a statistically significant difference in the number of cord-containing macrophages between granulomas from mice after one month and two months following infection (^∗∗^ *P* < 0.01, Figure 7(b)). No statistically significant difference was found in the number of granulomas with infected macrophages, the number of infected macrophages, or the number of colonies of replicating mycobacteria between granulomas from mice after one month and two months following infection* in vivo* (Figures 7(a) and 7(b)).

In Gran/1 m and Gran/2 m granulomas, the respective MOI counts were similar and so were the proportion counts; however, both counts were considerably higher in these granulomas than in Gran/20 d granulomas (^∗^ *P* < 0.05, Figure 7(c)). In the mice after one month and two months following infection, the respective numbers of granulomas with infected macrophages in them were similar (Figure 7(d)) and so were the respective numbers of infected macrophages (Figure 7(e)); however, both counts in these mice differed significantly from those in the mice after 20 days following infection. Thus, a considerable increase in the number of granulomas with macrophages containing two or more BCG-mycobacteria as well as in the number of macrophages with even more mycobacteria in them was observed at the end of the first month after BCG infection in the mice. Bacteriological determination of the number of mycobacteria in homogenates of various mouse organs and tissues [13, 45, 47] demonstrated that a logarithmic growth of mycobacterial populations during the first two weeks after infection generally reaches a plateau within the third and fourth weeks and is there for months during latent TB infection. Therefore, our data on the BCG load in mouse granulomas in chronic tuberculosis are consistent with the results obtained by other researchers.

## 4. Discussion

Studies of granulomas from mice with latent TB infection in* ex vivo* culture demonstrate that acid-fast BCG can be found in macrophages, dendritic cells, and multinucleate Langhans giant cells. The BCG-mycobacteria differed morphologically (in size and shape) and functionally (in replication rates) both in the same cell and in the granuloma cells of different types. A similar variability of* M. smegmatis* and* M. tuberculosis* cell growth and division during* in vitro* culture was demonstrated by Aldridge and colleagues [27] and that resulted in cell populations having different sensitivities to popular human antimycobacterial drugs.

In our study, BALB/c mice with latent TB infection differed both in the number of granulomas with infected cells they had and in the number of infected cells in granulomas they had. Furthermore, in the preparations obtained from each of the mice, granulomas had different sizes and contained different numbers of infected cells in them (from one to many more). No granulomas (not even the smallest) in which all the macrophages contained BCG were found. It is noteworthy that only acid-fast BCG-mycobacteria (which, again, have undamaged cell walls) were assayed in the host cells using the Ziehl-Neelsen method. These BCG-mycobacteria detected in the host cells of mouse granulomas probably remained in a metabolically active state, or so their capability of surviving and replicating in various cellular compartments suggests.

We have observed acid-fast BCG-mycobacteria in the visible vacuoles and apparently in the cytoplasm of mouse granuloma cells near the nuclei, which is where lysosomes normally get together, and in other cell compartments. However, no colocalization of LAM-labeled mycobacteria and acidic compartments was observed in splenic or bone marrow granuloma cells nor was it in peritoneal or bone marrow macrophages on day 20 following infection* in vivo*. The lack of colocalization of some mycobacteria and LysoTracker dye in various types of cells infected by different mycobacterial strains in* in vitro* culture has been pointed out by many researchers [22, 48–50]; however, this is the first time that this phenomenon is observed in the macrophages of individual granulomas isolated from mice with latent TB infection* in vivo*. The arrest of the biogenesis of phagolysosomes with mycobacteria engulfed in them is thought to represent one of the key mechanisms by which mycobacteria avoid host killing and survive within cells [4, 8, 10]. Therefore, further research is requited to elucidate the molecular mechanisms involved in the inhibition of phagosomal maturation in the cells of human and animal organisms during latent TB infection and after reactivation.

According to other studies [28, 29], various human and mouse cell lines infected* in vitro* rapidly eliminated attenuated mycobacteria (BCG) but did not eliminate virulent strains of* M. tuberculosis *or* M. bovis.* These studies demonstrated that some BCG-mycobacteria were killed by phagocytes in phagolysosomes and some died following apoptotic death of infected myeloid cells. However, in our study, we did not find macrophages or dendritic cells, with or without mycobacteria, that had a characteristic apoptotic morphology of cells in the* ex vivo* cultures of granulomas obtained from all BCG-infected mice. Furthermore, we have not found BCG-mycobacteria outside granuloma cells. It is possible that extracellular mycobacteria are absorbed by granuloma cells immediately, because, as was demonstrated previously [9], granuloma macrophages and dendritic cells in BCG-infected mice have an increased number of the phagocytic receptors CD11b, CD11c, CD14, and CD16/CD32, which typically show colocalization in microdomains on the surface of granuloma cells. We have also observed the replication of a large number of BCG-mycobacteria in the visible small and large vacuoles of granuloma macrophages, which suggests that acid-fast mycobacteria are not being eliminated in these compartments. This observation disagrees with the inference made from* in vitro* culture that mycobacteria can survive only in macrophage compartments, where close apposition between the mycobacterial surface and the host's phagosome membrane was determined [51]. It is possible that the differences in the behavior of microbes in macrophages were accounted for by the differences in the culture systems (*in vitro* versus* ex vivo*) and the mycobacterial strains (*M. avium* versus BCG) used. However, our data obtained from* ex vivo* culture suggest that actively replicating acid-fast mycobacteria were also present in the visible vacuoles of peritoneal and bone marrow macrophages after BCG infection* in vitro* (unpublished data).

A comparison of the granulomas obtained from spleens after one month and two months following infection did not actually reveal any difference in the number of infected granuloma cells. However, a considerable amount of individual variability for this number was observed across the BALB/c mice. It is possible that some mice were more sensitive to BCG infection than the others, and so they had more bacilli in their granuloma cells. By contrast, the others were capable of having BCG infection controlled, and so they had fewer mycobacteria in the cells of their granulomas. It is therefore important to understand what intercellular and intracellular interactions and what factors in the host organism and in granulomas themselves account for the varying sensitivity of the mice to BCG infection.

In our study, BCG cords in the cytoplasm of mouse granuloma macrophages and dendritic cells were observed for the first time. Mycobacterial cords are supposed to be analogs of biofilms that are dramatically resistant to antimicrobial agents [52]. Cord formation by mycobacteria had previously been observed mainly in* in vitro* culture of different strains of* M. tuberculosis* on solid and in liquid media or in the process of extracellular growth of* M. marinum* in zebrafish granulomas* in vivo* [31, 42, 43]. Low rates of BCG replication* in vitro* are a well-known fact [29]. We observed cords formed by BCG-mycobacteria in the host cells that were the two-day-old* ex vivo* cultures of granulomas (mice 8/2 m, 9/2 m, and 10/2 m) and assumed that those cords must have been formed earlier, in the mouse granuloma cells* in vivo*. In some studies [29, 53–55], the attenuated phenotype of BCG-mycobacteria and their inability to replicate and/or leave phagosomal vacuoles in host cells in cell lines following* in vitro* infection were explained by the absence, in the BCG genome, of some genes that virulent strains of* M. bovis* and* M. tuberculosis* possess and, first of all, by the absence of the RD1 locus containing the genes coding for proteins of the secretion system ESX-1. However, cording was observed in some granulomas of zebrafish infected with* M. marinum* with the deletion of the RD1 locus, when replicating mycobacteria were outside granuloma cells [31]. It had previously been established [12, 45] that cord formation by mycobacteria (including BCG) growing on solid and in liquid media* in vitro* might be controlled by the components of mycobacterial cell walls and, above all, by chemical modifications of mycolic acids. It was not known if the chemical modifications that account for cord formation were present in the cell walls of some BCG-mycobacteria in mouse granuloma cells. Probably, other mycobacteria did not have such chemical modifications of mycolic acids in the cell walls and represented groups of chaotically arranged replicating bacteria. These assumptions, however, require further studies.

Detection of granuloma macrophages with a large number of single BCG-mycobacteria, possibly in the cytoplasm of the host cells, raised the question as to how such host cells occur in mouse granulomas. It seemed possible that such cells could appear as a result of the replication of BCG-mycobacteria in the host cells and their subsequent migration from the colonies across the cells and/or between the cells. Another possible way of the occurrence of host cells with a large number of single BCG-mycobacteria in them is through phagocytosis of mycobacteria from the extracellular environment by granuloma macrophages followed by migration of mycobacteria from the phagosomes to the cytoplasm of the host cells, probably by lysis of vacuolar membranes. A similar mechanism of repeated infection of macrophages that engulf the necrotic cells containing mycobacteria has been revealed and studied in zebrafish granulomas at the initial stage of infection with* M. marinum* [39]. The migration of* M. marinum* from phagosomes to the cytoplasm of host cells and their traveling between granuloma macrophages with the help of actin and proteins bound to it have also been demonstrated [56–58]. In our experiments, we did not observe, in* ex vivo* culture, any granuloma macrophage or dendritic cell dead by necrosis, when BCG-mycobacteria could occur in extracellular locations after the host cells had been destroyed, or by apoptosis. According to some studies [28, 29], BCG-mycobacteria in human and mouse myeloid cell lines infected* in vitro* can only be found in phagosomes and phagolysosomes, which is where they are eliminated, while mycobacteria of virulent strains actively replicate in the vacuoles and/or the cytoplasm of the host cells. Some authors [29, 39, 55, 58–60] hypothesized that the processes of intracellular replication and intercellular dissemination of mycobacteria require the genetic locus known as RD1, which encodes the proteins ESAT-6 and GFP-10. These molecules are assumed to aid in phagosomal escape of virulent mycobacteria and their migration to the cytoplasm of host cells [29, 60]. However, our studies do not confirm this hypothesis: BCG-mycobacteria with the RD1 deletion [53] replicated actively in the visible vacuoles and presumably in the cytoplasm of granuloma host cells from mice with latent TB infection. It is possible that the mycobacteria behave differently in the host cells at the earliest stages of infection of macrophages* in vitro* and in granuloma cells obtained from mice after 20 days, one month, and two months following BCG infection* in vivo*. Further studies of the ways, factors, and mechanisms that influence the replication and dissemination of mycobacteria, including the vaccine strains, inside host cells, and between granuloma cells in mice with active and latent TB infection are required.

Importantly, the macrophages of the mouse granulomas being discussed retained their capacity of phagocytosis and ingestion of engulfed lymphocytes (with several features of apoptosis including nuclear fragmentation) and thrombocytes. At the same time, the mouse granuloma macrophages lacked bactericidal activity against BCG-mycobacteria that were located in the vacuoles and cytoplasm of the host cells with engulfed lymphocytes and thrombocytes. It has been noticed that the arrest of phagosome to phagolysosome maturation in granuloma macrophages was only confined to the compartments with BCG-mycobacteria inside. Our conclusions confirm the hypothesis proposed previously [10, 61] that those were the mycobacteria that are responsible for the ability to avoid degradation and survive in macrophage phagosomes. However, our study makes us disagree with the statement that mycobacterial infection of macrophages induces global modifications in the phagosomal functions of host cells that are observed in macrophages of various lines after infection* in vitro* [62]. It is noteworthy that vacuoles with BCG-mycobacteria in them do not seem to be static formations in granulomas from mice with latent TB infection. Rather, they are susceptible to change with the transition of mycobacteria from the vacuoles to the host cells' cytoplasm. The exact mechanisms of these processes are still not clear.

The macrophages of splenic and bone marrow granulomas obtained from mice after 20 days following BCG infection* in vivo* contained a lower number of acid-fast mycobacteria and the lowest number of replicating BCG-mycobacteria, as opposed to the granuloma cells of mice after one month and two months following BCG infection* in vivo*. At the same time, in our studies, the* in vitro* infection of mouse peritoneal and bone marrow macrophages with BCG resulted in a considerable growth of mycobacteria in the host cells after* in vitro* culture for 5 days (unpublished data). Therefore, the granuloma macrophages could control BCG infection in mice with latent tuberculosis* in vivo* and in the* ex vivo* culture. This could be true, because it has now and previously been demonstrated [9] that granuloma macrophages and dendritic cells of BCG-infected mice produce proinflammatory mediators, including the cytokines IFN*γ*, IL-1*α*, and GM-CSF. Moreover, most mycobacteria were efficiently eliminated in the hosts, as was shown by the mycobacterial counts in the peritoneal macrophages isolated from mice after 20 days following intraperitoneal infection with BCG* in vivo*. At this stage of latent tuberculosis, the dissemination of mycobacteria from the peritoneal cavity to other mouse organs, including the spleen, bone marrow, and lung, was already observed. Various numbers of solid granulomas were formed. Interestingly, acid-fast BCG-mycobacteria were found only in bone marrow macrophages, while it is only dendritic cells that are supposed to spread the microbes across the tissues of human and animal organisms so that miliary tuberculosis is eventually developed [8, 63, 64]. Thus, only a small number of BCG-mycobacteria survived in the granuloma cells and other host cells of mice after BCG infection* in vivo*. Identification of the differences in functional states that allow some of mycobacteria to reside and grow successfully in the hosts with increased production of the proinflammatory cytokines IFN*γ* and IL-1*α* in granuloma cells, while the others will be killed and eliminated, is a matter of further research.

As is known, BCG-mycobacteria are attenuated for man and are widely used in vaccine prophylaxis of tuberculosis in children [55]. As far as BALB/c mice are concerned, we have discovered a large number of relationships between different types of granuloma cells and BCG-mycobacteria. Within the same mouse line, these relationships range from almost complete absence of mycobacteria in granuloma macrophages from mice, whose organisms have neutralized the BCG infection (mouse 25 S/2 m), to active replication of mycobacteria, with the formation of cords, which is an indicator of mycobacterial virulence in the host cells and, probably, a marker of the activation of TB infection in animals (mice 3 S/1 m and 23 S/2 m). Therefore, this experimental mouse model is interesting for a preliminary assessment of the effect of antituberculosis drugs on mycobacteria in granuloma cells in animals with different MOIs, because, as was pointed out in a review by Franzblau and colleagues [35], good concordance was found between the results on the sensitivity of* M. bovis* BCG and* M. tuberculosis* to diverse compounds.

## 5. Conclusions

Our findings of the persistence of BCG-mycobacteria in macrophages, dendritic cells, and multinucleate Langhans giant cells of mouse granulomas, which have been studied for the first time, represents an interesting example of long-lasting specific interactions between macro- and microorganisms in chronic TB infection in animals. It is our hope that using an* ex vivo* monolayer cultures of granuloma cells in a mouse model of latent TB infection, relatively safe for the researchers, will be useful as a tool for exploring TB-causing relationships between intracellular pathogens and the host organisms and studying of various molecular and cellular mechanisms by which pathogenic microorganisms survive in animals and avoid numerous host-protecting mechanisms of the innate and the adaptive immune system.
